# Ketogenic Diet-Induced Weight Loss is Associated with an Increase in Vitamin D Levels in Obese Adults

**DOI:** 10.3390/molecules24132499

**Published:** 2019-07-09

**Authors:** Maria Perticone, Raffaele Maio, Angela Sciacqua, Edoardo Suraci, Angelina Pinto, Roberta Pujia, Roberta Zito, Simona Gigliotti, Giorgio Sesti, Francesco Perticone

**Affiliations:** 1 Department of Experimental and Clinical Medicine, University *Magna Græcia* of Catanzaro, 88100 Catanzaro, Italy; 2 Azienda Ospedaliero, Universitaria *Mater Domini* di Catanzaro, 88100 Catanzaro, Italy; 3 Department of Medical and Surgical Sciences, University *Magna Græcia* of Catanzaro, 88100 Catanzaro, Italy; 4 Department of Health Sciences, University *Magna Græcia* of Catanzaro, 88100 Catanzaro, Italy

**Keywords:** vitamin D, obesity, ketogenic diet

## Abstract

Vitamin D is an important micronutrient involved in several processes. Evidence has shown a strong association between hypovitaminosis D and cardio-metabolic diseases, including obesity. A ketogenic diet has proven to be very effective for weight loss, especially in reducing fat mass while preserving fat-free mass. The aim of this study was to investigate the effect of a ketogenic diet-induced weight loss on vitamin D status in a population of obese adults. We enrolled 56 obese outpatients, prescribed with either traditional standard hypocaloric Mediterranean diet (SHMD) or very low-calorie ketogenic diet (VLCKD). Serum 25(OH)D concentrations were measured by chemiluminescence. The mean value of serum 25-hydroxyvitamin D (25(OH)D) concentrations in the whole population at baseline was 17.8 ± 5.6 ng/mL, without differences between groups. After 12 months of dietetic treatment, in VLCKD patients serum 25(OH)D concentrations increased from 18.4 ± 5.9 to 29.3 ± 6.8 ng/mL (*p* < 0.0001), vs 17.5 ± 6.1 to 21.3 ± 7.6 ng/mL (*p* = 0.067) in the SHMD group (for each kilogram of weight loss, 25(OH)D concentration increased 0.39 and 0.13 ng/mL in the VLCKD and in the SHMD groups, respectively). In the VLCKD group, the increase in serum 25(OH)D concentrations was strongly associated with body mass index, waist circumference, and fatty mass variation. In a multiple regression analysis, fatty mass was the strongest independent predictor of serum 25(OH)D concentration, explaining 15.6%, 3.3%, and 9.4% of its variation in the whole population, in SHMD, and VLCKD groups, respectively. We also observed a greater reduction of inflammation (evaluated by high-sensitivity C reactive protein (hsCRP) values) and a greater improvement in glucose homeostasis, confirmed by a reduction of HOMA values, in the VLCKD versus the SHMD group. Taken together, all these data suggest that a dietetic regimen, which implies a great reduction of fat mass, can improve vitamin D status in the obese.

## 1. Introduction

Vitamin D is a fat-soluble steroid hormone involved in several physiologic activities, such as calcium homeostasis and musculoskeletal health [1,2]. Moreover, given the extensive tissue distribution of its receptors, vitamin D is also involved in processes of differentiation, cell proliferation, and hormone secretion [3]. Vitamin D is the generic term used for both vitamin D_2_ (ergocalciferol) and vitamin D_3_ (cholecalciferol). Vitamin D_2_ is synthesized by the exposure of ergosterol from plants to UVB radiation, while cholecalciferol is synthesized in the skin by the action of UVB radiation. Both vitamins D_2_ and D_3_ undergo a two-step hydroxylation process. Firstly, vitamins D_2_ and D_3_ undergo a first hydroxylation step in the liver, were they are converted into 25(OH)D_2_ and 25(OH)D_3_, respectively. In the second step of the hydroxylation process, 25(OH)D_2_ and 25(OH)D_3_ are converted to the biologically active form of vitamin D (1,25(OH)_2_D) in the kidney [4]. Some evidence indicates a strong association between hypovitaminosis D and different cardio-metabolic diseases [5,6,7,8], suggesting the hypothesis that vitamin D deficiency may play a crucial role in the pathogenesis of these conditions. In particular, suboptimal serum vitamin D concentrations may have a causative role in the appearance of impaired glucose homeostasis, insulin resistance, and type-2 diabetes [9,10,11]. On the contrary, more recent evidence demonstrated a causal role of obesity in determining vitamin D deficiency [12]. Vitamin D status is evaluated through the measurement of serum concentrations of 25-hydroxyvitamin D (25(OH)D); values below 20 ng/mL should be considered as a deficiency of this micronutrient [13].

Several studies have identified that obesity is associated with vitamin D deficiency [12,14], although further studies are needed to determine whether obesity-related vitamin D status affects obesity-related health risks [15]. It is well-established that serum 25(OH)D is about 20% lower in obese people than normal weight [16], and the prevalence of 25(OH)D deficiency is greater in obese populations [17]. Furthermore, 25(OH)D concentrations are inversely correlated with body weight, body mass index (BMI), and fat mass (FM) [18]. It has been demonstrated that vitamin D supplementation has no effect on body weight or fat mass [19]. The proposed mechanisms underlying vitamin D deficiency in obese patients are: (a) a lower dietary intake, (b) lower sunlight exposure or impaired skin synthesis of vitamin D, and (c) 25(OH)D distribution into a larger whole-body tissue volume [20]. The latter hypothesis seems to be the most accepted, since recent data demonstrated that the large mass of adipose tissue in obesity forms an enlarged vitamin D reservoir [21]. 

A ketogenic diet (KD) is one of the most effective diet regimes that has proven to be very effective for rapid weight loss. This approach recognizes the predominant source of energy in ketone bodies deriving from lipid metabolism when carbohydrates are absent or low [22]. High-fat KD was used in 1921 to treat epilepsy because it was documented that abnormally high levels of ketone bodies ameliorated seizures [23]; this kind of KD is known as “standard KD”. A modified KD, characterized by a low intake of both carbohydrates and lipids, known as very low-calorie (<800 kcal per day) ketogenic diet (VLCKD), is used as an effective regimen for weight loss [24] because of the ability of ketone bodies to suppress appetite, allowing a very small calorie intake. Another important feature of KD is the fact that, thanks to an adequate protein intake (0.8–1.5 g per kilogram of ideal body weight), lean mass is preserved. 

Thus, we designed the present study to investigate if weight loss obtained through VLCKD is associated with an increase in serum 25(OH)D concentration.

## 2. Results

### 2.1. Study Population

The whole study population was divided into two groups according to the type of diet prescribed: VLCKD and SMHD (standard hypocaloric Mediterranean diet) groups. Baseline demographic, anthropometric, clinical, and biochemical characteristics of the whole study population and of the two groups separately are reported in Table 1.

There were no significant differences between groups for any of the considered parameters, except for gender distribution (*p* = 0.036), HbA1c (*p* = 0.013), and diastolic blood pressure (DBP) (*p* = 0.029). As expected, serum 25(OH)D concentrations were low in both groups, without significant difference.

In Table 2 and in Table 3 we report comparisons of anthropometric, clinical, biochemical, and hemodynamic characteristics before and after 12 months of dietary treatment in VLCKD and SHMD groups, respectively (in the SHMD group, four patients were lost to follow-up). Regarding anthropometric parameters, patients in the VLCKD group showed a clinically, even if not statistically, significant reduction in both body weight (Δ= −26.6 kg, *p* = 0.097) and BMI (Δ= −7.2 kg/m^2^, *p* = 0.212), passing from third- to the first-grade obesity [25]. They also showed a significant reduction in waist circumference (WC). Total body water (TBW) significantly increased after 12 months of VLCKD, probably reflecting the changes in body composition, especially the reduction in FM. When considering the biochemical parameters, VLCKD patients showed a significant improvement in HbA1c, fasting insulin, homeostasis model assessment (HOMA), triglycerides, high-sensitivity C reactive protein (hsCRP), and 25(OH)D. On the contrary, in patients in the SHMD group, we did not observe any significant improvement in anthropometric parameters. In the same group, when considering biochemical parameters, we observed a significant improvement only in the following: fasting plasma glucose, HbA1c, fasting insulin, HOMA, triglycerides, and hsCRP.

Interestingly, for each kilogram of weight loss, 25(OH)D concentration increased 0.39 and of 0.13 ng/mL in VLCKD and SHMD groups, respectively.

When considering dietary adherence, 95% of patients in the VLCKD group showed a good compliance to the prescribed dietary regimen, while in SHMD, four patients were lost to follow-up, and only 55% of the remaining patients achieved an acceptable degree of adherence. 

### 2.2. Correlational Analysis

A linear regression analysis was performed to test the relationship between Δ25(OH)D and different covariates in the study population and in the two groups separately (Table 4).

In the whole study population, Δ25(OH)D was significantly correlated with the following anthropometric variables: ΔBMI (r = 0.425) and ΔWC (r = 0.338). When considering biochemical parameters, a significant, linear correlation was found between Δ25(OH)D and the following: total cholesterol (r = 0.065), low-density lipoprotein (LDL)-cholesterol (r = 0.104), triglycerides (r = 0.056), HOMA (r = 0.312), and hsCRP (r = 0.171), while an inverse relationship was found with high-density lipoprotein (HDL)-cholesterol (r = −0.175). Regarding hemodynamic parameters, only diastolic blood pressure (DBP) reached statistical significance (r = 0.219).

In the VLCKD group, a strong linear relationship was found between Δ25(OH)D and the following anthropometric covariates: ΔBMI (r = 0.337), ΔWC (r = 0.219), and ΔFM (r = 0.123). A significant correlation was also found between Δ25(OH)D and the following biochemical and hemodynamic parameters: total cholesterol (r = 0.127), triglycerides (r = 0.098), HOMA (r = 0.249), hsCRP (r = 0.076), and systolic blood pressure (SBP) (r = 0.220).

In the SHMD group, Δ25(OH)D correlated significantly only with ΔBMI (r = 0.165), ΔWC (r = 0.178), and HOMA (r = 0.369). 

### 2.3. Multivariate Analysis

A stepwise multivariate regression analysis was performed to evaluate the independent predictors of Δ25(OH)D in the whole study population and in the two groups separately (Table 5), also including gender and smoking as independent covariates. 

In the whole population, as well as in the VLCKD and in the SHMD groups, ΔFM was the major predictor of Δ25(OH)D, explaining 15.0%, 12.1%, and 9.6% of variations, respectively. In the whole population, other independent predictors were ΔBMI, ΔWC, and HOMA, explaining another 7.2%, 3.6%, and 1.1% of Δ25(OH)D variation, respectively. 

In the VLCKD group, other independent predictors of Δ25(OH)D variations were ΔBMI and ΔWC, explaining, respectively, another 6.9% and 4.5% of its variation. In the SHMD group, ΔBMI explained another 2.8% of Δ25(OH)D variation.

## 3. Discussion

The results of this study demonstrate, for the first time, that weight loss obtained through a long-lasting VLCKD regimen is associated with an increase in serum 25(OH)D concentrations, while no significant difference is observed in the SHMD group. Interestingly, for each kilogram of weight loss, 25(OH)D concentration increased 0.39 and of 0.13 ng/mL in VLCKD and SHMD groups, respectively. These findings are in contrast with previously published literature, which suggested a negative effect of VLCKD on bone composition, because ketoacidosis generated by low-carbohydrate/high-protein diets results in hypercalciuria [26] consequent to the renal compensatory response to the dietary acid challenge. In turn, the skeleton supplies serum buffer by active reabsorption of bone, thereby leading to hypercalciuria and an adverse effect on bone quality. It has been demonstrated that dietary regimens rich in animal protein may exert deleterious effects on bone quality in rats, but less is known about the effect of low-carbohydrate diets in humans. In fact, the majority of the studies demonstrating a loss of bone mineral content in subjects following ketogenic diets were conducted in children following a low-carbohydrate, high-fat regimen [27,28]. On the other hand, Colica et al. demonstrated that VLCKDs did not cause negative changes in global measurements of nutritional state including sarcopenia, bone mineral content, and hepatic, renal, and lipid profiles as well as an increase vitamin D levels in the short term [29].

Interestingly, we observed that patients in the VLCKD group showed greater weight loss compared to the SHMD group after 12 months of treatment. In our opinion, this could be attributable to the lower dietary adherence observed in the SHMD group.

Of interest, our data demonstrate that, in the VLCKD group, the increase in serum 25(OH)D concentration was strongly associated with ΔBMI, ΔWC, and ΔFM. A significant correlation was also found between Δ25(OH)D and the following biochemical and hemodynamic variables: total cholesterol, triglycerides, HOMA, hsCRP, and SBP. Moreover, in the stepwise multivariate regression analysis, ΔFM was the major predictor of Δ25(OH)D in the whole study population as well as in the VLCKD and SHMD groups, explaining 15.0%, 12.1%, and 9.6% of its variation, respectively. 

Another important finding in this study is the improvement in glucose homeostasis parameters in both groups after 12 months of nutritional therapy, with the most impressive improvement observed in the VLCKD group. Of note, HOMA was another independent predictor of the variation of serum 25(OH)D concentrations after weight loss in the whole study population, confirming previously published data, which demonstrated that vitamin D status affects glucose and insulin homeostasis [30].

The results of this study also demonstrate a greater reduction of inflammation (evaluated by hsCRP values) in patients who underwent a VLCKD regimen compared to patients who followed a SHMD regimen. This finding confirms previously published data [31], in which VLCKD was associated with a reduction of inflammatory variables. 

Taken together, these findings suggest that weight loss obtained through VLCKD might be useful in improving vitamin D status in obese patients through a greater reduction in FM, compared with a SHMD regimen. This might have a positive impact on the reduction of the cardio-metabolic risk related to hypovitaminosis D.

In conclusion, the most relevant strength of this study is that a long-lasting VLCKD, with a consequent marked loss of FM, is a powerful therapeutic approach to improve vitamin D status, glucose homeostasis, and inflammatory profile in obese patients. This study has some potential limitations. The first limitation is represented by the small sample size; more data are needed on a larger population. Secondly, the duration of follow-up was short; our data need to be confirmed in longer trials. Thirdly, the laboratory determinations lack in some biomarkers (i.e., ketone bodies, cytokines, etc.), and we did not perform an analysis of bone mineral content. 

## 4. Materials and Methods 

### 4.1. Study Population

**Figure 1:**
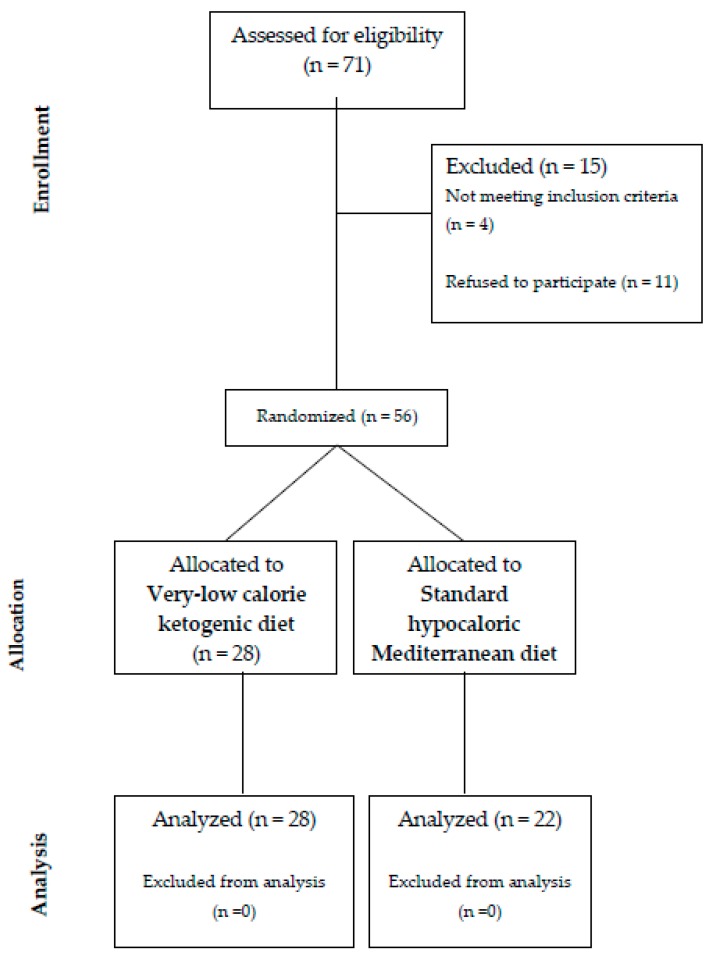
CONSORT diagram.

For this controlled, open design study, between December 2016 and May 2017 we enrolled 56 obese outpatients (32 males and 24 females; mean age 46.75 ± 11.05 years) referred to our University Hospital for an evaluation of cardiovascular risk factors. Patients were randomized and allocated to receive either a VLCKD or a SHMD on the basis of their dietary preferences and nutritional requirements (Figure 1). Inclusion criteria were: age > 18 years and BMI > 30 kg/m^2^. Exclusion criteria were: pregnancy, breastfeeding, type-1 diabetes mellitus, heart failure, history or clinical evidence of angina, myocardial infarction, valvular heart disease, neoplastic disease, estimated glomerular filtration rate (eGFR) < 60 mL/min/1.73 m^2^, liver dysfunction (an increase of at least two-fold above the upper limit in GOT and GPT), use of medications able to interfere with glucose and/or vitamin D metabolism, thyroid disorders, and history or clinical evidence of psychiatric disorders. Eligible patients were divided into two groups (VLCKD group and SHMD). All evaluations were performed at baseline and after 12 months of dietary treatment, avoiding problems from seasonal variation in the time of study.

The local Ethics Committee approved the study and the related informed consent. Subjects eligible for the study gave their informed written consent prior to entry, and all study procedures were performed in accordance with the Declaration of Helsinki.

### 4.2. Anthropometric Measurements

Anthropometric measurements for all participants were carried out at each visit between 8:00 and 9:00 a.m. according to a standardized protocol [32]. All measurements were performed with subjects without clothes and shoes.

Waist and hip circumferences were measured using a flexible metric tape to the nearest 0.5 cm. WC was taken just above the iliac crest, while hip circumference was measured at the greatest posterior protuberance of the buttocks. Body weight was measured using an electronic balance scale, and height was taken using a stadiometer to the nearest 0.1 cm. BMI was calculated using the following formula: BMI = body weight (kg)/height (m^2^).

### 4.3. Laboratory Determinations

All laboratory measurements were performed after a fast of at least 12 h. Plasma glucose was determined immediately by the glucose oxidase method (Glucose Analyzer, Beckman Coulter S.p.A., Milan, Italy). Triglycerides and total LDL- and HDL-cholesterol concentrations were measured by standard enzymatic methods (Roche Diagnostics GmbH, Mannheim, Germany). Serum insulin was determined in duplicate by a highly specific radioimmunoassay using two monoclonal antibodies: intra-assay coefficient of variation (CV) 2.1% and inter-assay CV 2.9%. Insulin resistance (IR) was estimated by HOMA-IR according to the following equation: HOMA = [insulin (μU/mL) * glucose (mmol/L)]/22.5 [33].

In patients in the VLCKD group, ketosis was measured daily using ketone urine testing strips (Bayer Ketositx®, Ascensia Diabetes Care, Basel, Switzerland) at 8:00 a.m. after overnight fast.

hsCRP was measured by a high-sensitivity turbidimetric immunoassay (Behring, Marburg, Germany). Values of e-GFRwere calculated by using the new equation proposed by investigators in the Chronic Kidney Disease Epidemiology (CKD-EPI) Collaboration [34].

Serum concentrations of 25(OH)D were measured by direct competitive immunoassay with chemiluminescence (CLIA) through the Liaison–Diasorin test, in all patients. Venous samples for the determination of 25(OH)D were carried out in the morning on fasting conditions. A sample of 2.5 mL of venous blood was collected and subsequently introduced into a disposable test tube equipped with separation accelerator granules. The blood sample was centrifuged for 5 min at 2500 rpm in a special centrifuge, and 500 μL of the obtained serum was collected and placed in special cuvettes. During the first incubation, 25(OH)D was dissociated from its binding protein and tied to the specific antibody. After 10 min, a tracer consisting of vitamin D linked to an isoluminol derivative was added and incubated for another 10 min. Subsequently, the unbound material was removed with a wash cycle. In the last step, starter reagents for the chemiluminescence reaction were added to initiate a flash chemiluminescent reaction. Finally, the light signal was measured by a photomultiplier as relative light units (RLUs), and it was inversely correlated with 25 (OH)D concentrations in each sample. Quality control was performed by BioRad Liquicheck Specialty Immunoassay Controls Level 1 and Level 2 on each day that samples were analyzed. Data on the cross-reactivity of the antiserum used in this assay were obtained by spiking the potential cross-reactant up to 100 ng/mL and assaying. The cross-reactivity of each compound, normalized to 25-OH vitamin D_3_, is listed in the manufacturer’s manual. Vitamin D levels were classified as follows: deficient (<20 ng/mL), insufficient (20 to <30 ng/mL), and normal (≥30 ng/mL). 

### 4.4. Body Composition Assessment

A single-frequency bioelectrical impedance assessment (BIA) was performed in the morning after 10 min rest in the supine position. Impedance was measured by one single measurement of resistance (in Ohms) and reactance (in Ohms) with BIA-101 equipment (Akern Florence, Italy). Short- and long-term reliability of resistance measurements indicated coefficients of variation between 2.7% and 4.0% [31]. The four electrodes were attached on the dorsal side of the foot, on the ankle, on the dorsal side of the hand, and on the wrist at the right side of the body. FFM was calculated using manufacturer-supplied equations based on a comparison with densitometry in a normal population. 

### 4.5. Dietary Treatment

According to the European Food Safety Authority (EFSA), VLCKDs are formulated foods that, when used as instructed by the manufacturer, replace the whole diet (total meal replacement) and provide less than the minimum amount of energy provided by low-calorie diets. In particular, VLCKD is characterized by an energy intake of 600 kcal per day with 50%–60% of energy intake derived from proteins, 20%–30% from lipids, and 20% from carbohydrates [35]. All nutritional requirements were met using five to six formulated meals a day containing about 15–18 g of high biological value protein preparations, 4 g carbohydrates, and 3 g fat. The weight-loss program consisted of five steps; the first three steps consisted of a VLCKD (600–800 kcal/day) low in carbohydrates (<50 g daily, derived from vegetables) and lipids (10 g of olive oil/day). In step 1, patients were prescribed five to six protein preparations/day, vegetables, and olive oil. In step 2, one of the formulated meals was substituted with either 180 g of fresh meat or fish or 2 eggs either at lunch or at dinner. In step 3, a second serving of formulated meals was substituted with a second serving of fresh meat or fish. During these steps a capsule of multivitamins, proper integration of mineral salts, and an alkalizing product were prescribed to all patients. These three steps were maintained until the patient lost about 80% of the target amount of weight, and the length of these phases depended on the weight loss target. Then, in steps 4 and 5, patients started a low-calorie diet (1000–1500 kcal/day) with progressive incorporation of different food groups. When patients reached the target weight, they underwent a maintenance diet (1500–2000 kcal/day).

Patients in SHMD were prescribed a Mediterranean diet with a caloric deficit of 500 kcal/day based on basal metabolic rate (BMR). The dietetic program was characterized by 55%–60% of energy intake derived from carbohydrates, 10%–15% from proteins, and 25%–30% from lipids [36]. Patients in this group followed a balanced diet allowing the use of whole grain pasta, bread, rice, meat, fish, eggs, and vegetables in different combinations, as prescribed by an experienced dietitian.

### 4.6. Dietary Adherence 

Adherence to the diet was determined through self-reports and food records in both groups. In addition, in the VLCKD group we also performed urinary ketone qualitative assessments at each hospital visit (monthly).

Furthermore, during all visits, patients were asked to complete a questionnaire regarding side effects and dropouts, and the reasons provided were recorded. At 3, 6, and 12 months, a questionnaire on patient satisfaction with their diet was administered, and the data were recorded. 

### 4.7. Statistical Analysis 

ANOVA for continuous clinical and biological data was used to test for the differences among groups, and the Bonferroni post hoc test for multiple comparisons was further performed. For dichotomic variables we used the chi-square test. Data were expressed as mean ± standard deviation, and binary data as percent frequency. The Pearson method was used to calculate the relationship between the variation of 25(OH)D after 12 months of dietary treatment and the following covariates: age; systolic blood pressure (SBP); DBP; total, LDL-, and HDL-cholesterol; triglycerides; HOMA; and hsCRP in both groups. We also considered as covariates the difference in values (Δ) between baseline and 12 months for the following parameters: BMI, WC, FM, free-fat mass (FFM), and muscle mass (MM). Variables that reached statistical significance (gender and smoking as dichotomous values) were inserted in a stepwise multivariate linear regression model to determine the independent predictors of Δ25(OH)D, and these analyses were performed for the whole study population and for the two groups separately. Differences were assumed to be significant at P < 0.05. All comparisons were performed using SPSS 20.0 statistical software (SPSS, Inc., Chicago, IL, USA).

## Figures and Tables

**Table 1:** Baseline demographic, anthropometric, clinical, biochemical, and hemodynamic characteristics of the whole study population and of the two groups separately.

	All *n* = 56	SHMD *n* = 28	VLCKD *n* = 28	*p* value
**Age**, *years*	46.8 ± 11.0	50.9 ± 13.3	42.6 ± 6.6	0.090
**Sex**, *M/F*	32/24	18/10	14/14	0.036
**Body weight**, *Kg*	114.1 ± 28.8	107.5 ± 18.5	113.9 ± 31.0	0.307
**BMI**, *Kg/m^2^*	39.65 ± 9.7	38.8 ± 4.5	40.5 ± 10.8	0.307
**WC**, *cm*	123.0 ± 18.7	126.4 ± 12.2	119.1 ± 22.9	0.761
**HC**, *cm*	127.2 ± 9.6	125.2 ± 7.8	129.4 ± 8.3	0.098
**TBW**, *l*	44.6 ± 12.8	42.6 ± 9.8	48.7 ± 14.8	0.463
				
**FM**, *Kg*	49.3 ± 19.9	47.0 ± 10.8	51.3 ± 26.9	0.361
**FFM**, *Kg*	57.6 ± 14.7	56.1 ± 12.8	59.1 ± 16.9	0.647
**MM**, *Kg*	40.8 ± 12.7	38.2 ± 11.5	43.5 ± 13.9	0.354
**BMR**, *Kcal*	1818 ± 298	1818 ± 286	1820 ± 343	0.953
**FPG**, *mg/dL*	112.4 ± 29.9	115.3 ± 32.6	105.8 ± 25.5	0.819
**HbA1c**, *%*	6.3 ± 1.6	6.5 ± 1.5	6.1 ± 1.4	0.013
**Fasting insulin**, *μU/L*	26.9 ± 14.5	25.8 ± 15.4	28.0 ± 16.7	0.509
**HOMA**	7.4 ± 0.5	7.4 ± 0.9	7.3 ± 0.7	0.665
**eGFR**, *ml/min/1,73m^2^*	104.9 ± 11.0	103.7 ± 11.0	106.0 ± 12.2	0.617
**Uric acid**, *mg/dL*	5.5 ± 1.3	5.4 ± 1.3	5.6 ± 1.0	0.814
**Total cholesterol**, *mg/dL*	184.9 ± 34.1	180.7 ± 40.7	192.3 ± 27.0	0.597
**HDL cholesterol**, *mg/dL*	51.7 ± 13.3	51.1 ± 15.4	52.2 ± 11.0	0.668
**LDL cholesterol**, *mg/dL*	121.4 ± 33.7	115.3 ± 43.9	126.9 ± 20.5	0.408
**Triglycerides**, *mg/dL*	155.0 ± 94.0	158.5 ± 62.3	151.3 ± 50.0	0.706
**hsCRP**, *mg/L*	5.05 ± 3.5	5.6 ± 4.3	4.5 ± 2.6	0.943
**SBP**, *mmHg*	129.6 ± 18.1	123.4 ± 16.9	132.8 ± 16.3	0.129
**DBP**, *mmHg*	79.7 ± 12.3	73.5 ± 8.9	83.1 ± 11.5	0.029
**25(OH)D**, *ng/ml*	17.8 ± 5.6	17.5 ± 6.1	18.4 ± 5.9	0.567

Data are expressed as mean ± standard deviation. **25(OH)D** = 25-hydroxyvitamin D; **BMI** = body mass index; **BMR** = basal metabolic rate; **DBP** = diastolic blood pressure; **eGFR** = estimated glomerular filtration rate; **FM** = fat mass; **FFM** = fat-free mass; **FPG** = fasting plasma glucose; **HC** = hip circumference; **HOMA** = homeostasis model assessment; **hsCRP** = high-sensitivity C reactive protein; **MM** = muscle mass; **SBP** = systolic blood pressure; **SHMD** = standard Mediterranean hypocaloric diet; **TBW** = total body water; **VLCKD** = very low-calorie ketogenic diet; and **WC** = waist circumference.

**Table 2:** Comparisons of the anthropometric, clinical, biochemical, and hemodynamic characteristics of patients in the whole VLCKD group at baseline and after 12 months of dietary treatment.

	VLCKD (*n* = 28)T0	VLCKD (*n* = 28) T12	*p* Value
***Anthropometric***			
**Body weight**, *Kg*	113.9 ± 31.0	87.3 ± 22.8	0.097
**BMI**, *Kg/m^2^*	40.5 ± 10.8	33.3 ± 9.72	0.212
**WC**, *cm*	119.1 ± 22.9	95.0 ± 17.4	0.044
**HC**, *cm*	129.4 ± 8.3	118.5 ± 7.3	0.066
**TBW**, *l*	48.7 ± 14.8	55.3 ± 13.9	0.035
**FM**, *Kg*	51.3 ± 16.9	31.4 ± 8.6	0.141
**FFM**, *Kg*	59.1 ± 16.9	62.7 ± 15.6	0.835
**MM**, *Kg*	43.5 ± 13.9	45.5 ± 15.6	0.760
**BMR**, *Kcal*	1820 ± 343	1646 ± 180	0.280
***Biochemical***			
**FPG**, *mg/dL*	105.8 ± 25.5	93.5 ± 13.7	0.304
**HbA1c**, *%*	6.1 ± 1.4	5.2 ± 0.15	0.022
**Fasting insulin**, *μU/L*	28.0 ± 16.7	10.9 ± 4.9	0.032
**HOMA**	7.3 ± 0.7	2.6 ± 0.2	<0.0001
**eGFR**, *ml/min/1,73m^2^*	106.0 ± 12.2	109.7 ± 11.1	0.563
**Uric acid** *, mg/dL*	5.6 ± 1.0	4.9 ± 0.8	0.257
**Total cholesterol**, *mg/dL*	192.3 ± 27.0	172.7 ± 21.0	0.158
**HDL cholesterol**, *mg/dL*	52.2 ± 11.0	56.5 ± 13.6	0.515
**LDL cholesterol**, *mg/dL*	126.9 ± 20.5	109.3 ± 29.6	0.077
**Triglycerides**, *mg/dL*	151.3 ± 50.0	72.3 ± 29.6	0.004
**hsCRP**, *mg/L*	4.5 ± 2.6	1.8 ± 0.8	<0.0001
**25(OH)D**, *ng/mL*	18.4 ± 5.9	29.3 ± 6.8	<0.0001
***Hemodynamic***			
**SBP**, *mmHg*	132.8 ± 16.3	118.8 ± 15.1	0.118
**DBP**, *mmHg*	83.1 ± 11.5	79.3 ± 4.8	0.463

Data are expressed as mean ± standard deviation. **25(OH)D** = 25-hydroxyvitamin D; **BMI** = body mass index; **BMR** = basal metabolic rate; **DBP** = diastolic blood pressure; **eGFR** = estimated glomerular filtration rate; **FM** = fat mass; **FFM** = fat-free mass; **FPG** = fasting plasma glucose; **HC** = hip circumference; **HOMA** = homeostasis model assessment; **hsCRP** = high-sensitivity C reactive protein; **MM** = muscle mass; **SBP** = systolic blood pressure; **TBW** = total body water; **VLCKD** = very low-calorie ketogenic diet; and **WC** = waist circumference.

**Table 3:** Comparisons of the anthropometric, clinical, biochemical, and hemodynamic characteristics of patients in the whole SHMD group at baseline and after 12 months of dietary treatment.

	SHMD (*n*= 28) T0	SHMD (*n*= 22) T12	*p* Value
***Anthropometric***			
**Body weight**, *Kg*	107.5 ± 18.5	99.3 ± 15.8	0.254
**BMI**, *Kg/m^2^*	38.8 ± 4.5	36.1 ± 5.7	0.321
**WC**, *cm*	126.4 ± 12.2	113.0 ± 14.5	0.098
**HC**, *cm*	125.2 ± 7.8	119.5 ± 5.6	0.665
**TBW**, *l*	42.6 ± 9.8	42.9 ± 8.9	0.754
**FM**, *Kg*	47.0 ± 10.8	40.5 ± 8.6	0.181
**FFM**, *Kg*	56.1 ± 12.8	55.3 ± 11.5	0.766
**MM**, *Kg*	38.2 ± 11.5	39.7 ± 11.7	0.840
**BMR**, *Kcal*	1818 ± 286	1730 ± 166	0.954
***Biochemical***			
**FPG**, *mg/dL*	115.3 ± 32.6	99.7 ± 11.4	0.048
**HbA1c**, *%*	6.5 ± 1.5	5.4 ± 0.18	0.034
**Fasting insulin**, *μU/L* **HOMA**	25.8 ± 15.4 7.4 ± 0.9	13.5 ± 6.7 3.5 ± 0.4	0.044 0.001
**eGFR**, *ml/min/1,73m^2^*	103.7 ± 11.0	105.7 ± 11.7	0.563
**Uric acid**, *mg/dL*	5.4 ± 1.3	5.6 ± 1.0	0.776
**Total cholesterol**, *mg/dL*	180.7 ± 40.7	168.6 ± 34.6	0.438
**HDL cholesterol**, *mg/dL*	51.1 ± 15.4	52.3 ± 15.4	0.685
**LDL cholesterol**, *mg/dL*	115.3 ± 43.9	104.9 ± 27.8	0.546
**Triglycerides**, *mg/dL*	158.5 ± 62.3	113.0 ± 21.5	0.039
**hsCRP**, *mg/L*	5.6 ± 4.3	3.7 ± 1.2	0.044
**25(OH)D**, *ng/mL*	17.5 ± 6.1	18.6 ± 6.8	0.645
***Hemodynamic***			
**SBP**, *mmHg*	123.4 ± 16.9	119.8 ± 15.1	0.063
**DBP**, *mmHg*	73.5 ± 8.9	72.2 ± 7.6	0.067

Data are expressed as mean ± standard deviation. **25(OH)D** = 25-hydroxyvitamin D; **BMI** = body mass index; **BMR** = basal metabolic rate; **DBP** = diastolic blood pressure; **eGFR** = estimated glomerular filtration rate; **FM** = fat mass; **FFM** = fat-free mass; **FPG** = fasting plasma glucose; **HC** = hip circumference; **HOMA** = homeostasis model assessment; **hsCRP** = high-sensitivity C reactive protein; **MM** = muscle mass; **SBP** = systolic blood pressure; **SHMD** = standard hypocaloric Mediterranean diet; **TBW** = total body water; and **WC** = waist circumference.

**Table 4:** Linear regression analysis between Δ25(OH)D and different covariates in the whole population and in the two groups separately.

	All (*n* = 56) r/P	VLCKD (*n* = 28) r/P	SHMD (*n* =24) r/P
***Anthropometric***			
**Age**, *years*	0.250/0.334	0.305/0.221	0.088/0.187
**ΔBMI**, *Kg/m^2^*	0.425/<0.0001	0.337/<0.0001	0.165/0.007
**ΔWC**, *cm*	0.338/<0.0001	0.219/<0.0001	0.178/0.005
**ΔFM**, *kg*	0.276/0.008	0.123/<0.0001	0.087/0.052
**ΔFFM**, *kg*	0.096/0.123	0.065/0.098	0.052/0.234
**ΔMM**, *kg*	−0.111/0.164	−0.094/0.078	−0.045/0.198
***Biochemical***			
**Total cholesterol**, *mg/dL*	0.065/0.043	0.127/0.020	0.099/0.326
**LDL-cholesterol**, *mg/dL*	0.104/0.047	0.153/0.139	0.039/0.346
**HDL-cholesterol**, *mg/dL*	−0.175/0.002	−0.101/0.237	−0.107/0.139
**Triglycerides**, *mg/dL*	0.056/0.002	0.098/<0.0001	0.128/0.098
**HOMA**	0.312/<0.0001	0.249/<0.0001	0.369/0.004
**hsCRP**, *mg/L*	0.171/0.003	0.076/0.007	0.034/0.09
***Hemodynamic***			
**SBP**, *mmHg*	0.331/0.067	0.220/0.043	0.245/0.544
**DBP**, *mmHg*	0.219/0.007	0.154/0.138	0.138/0.444

*r=* coefficient of variation; P= Pearson’s coefficient; **BMI** = body mass index; **DBP** = diastolic blood pressure; **FM** = fat mass; **FFM** = fat-free mass; **HOMA** = homeostasis model assessment; **hsCRP** = high-sensitivity C reactive protein; **MM** = muscle mass; **SBP** = systolic blood pressure; and **WC** = waist circumference.

**Table 5:** Stepwise multivariate regression analysis of Δ25(OH)D and different covariates in the whole population and in the two groups separately.

	*r^2^* partial	*r^2^* total	P
**All**			
ΔFM, *kg*	15	15	<0.0001
ΔBMI, *kg/m^2^*	7.2	22.2	<0.0001
ΔWC, *cm*	3.6	25.8	0.007
HOMA	1.1	26.9	0.002
**VLCKD**			
ΔFM, *kg*	12.1	12.1	<0.0001
ΔBMI, *kg/m^2^*	6.9	19.0	0.009
ΔWC, *cm*	4.5	23.5	0.001
**SHMD**			
ΔBMI, *kg/m^2^*	9.6	9.6	<0.0001
ΔFM, *kg*	2.8	12.4	0.04

*r^2^*= coefficient of determination. **BMI** = body mass index; **FM** = fat mass; **FFM** = fat-free mass; **HOMA** = homeostasis model assessment; **hsCRP** = high-sensitivity C reactive protein; **and MM** = muscle mass.
